# Influence of Surface Properties on Adhesion Forces and Attachment of* Streptococcus mutans* to Zirconia* In Vitro*

**DOI:** 10.1155/2016/8901253

**Published:** 2016-11-15

**Authors:** Pei Yu, Chuanyong Wang, Jinglin Zhou, Li Jiang, Jing Xue, Wei Li

**Affiliations:** State Key Laboratory of Oral Diseases, West China School of Stomatology, Sichuan University, Chengdu, Sichuan, China

## Abstract

Zirconia is becoming a prevalent material in dentistry. However, any foreign bodies inserted may provide new niches for the bacteria in oral cavity. The object of this study was to explore the effect of surface properties including surface roughness and hydrophobicity on the adhesion and biofilm formation of* Streptococcus mutans* (*S. mutans*) to zirconia. Atomic force microscopy was employed to determine the zirconia surface morphology and the adhesion forces between the* S. mutans* and zirconia. The results showed that the surface roughness was nanoscale and significantly different among tested groups (*P* < 0.05): Coarse (23.94 ± 2.52 nm) > Medium (17.00 ± 3.81 nm) > Fine (11.89 ± 1.68 nm). The contact angles of the Coarse group were the highest, followed by the Medium and the Fine groups. Increasing the surface roughness and hydrophobicity resulted in an increase of adhesion forces and early attachment (2 h and 4 h) of* S. mutans* on the zirconia but no influence on the further development of biofilm (6 h~24 h). Our findings suggest that the surface roughness in nanoscale and hydrophobicity of zirconia had influence on the* S. mutans* initial adhesion force and early attachment instead of whole stages of biofilm formation.

## 1. Introduction

Due to the great mechanical properties, biocompatibility, and excellent esthetic properties, zirconia has been widely applied for the fabrication of crowns, bridges, and ceramic posts in dentistry. Also it has been introduced to orthodontics and dental implantology in order to improve esthetic effect [1, 2]. However, any foreign bodies inserted in oral cavity may provide new niches for the microorganisms, promoting biofilm accumulation. Such biofilm formation on dental materials appears to be similar to that around natural tooth, which potentially contributes to damage to the mineralized tissues or infections of the soft tissues. Enamel demineralization caused by biofilm formation near the bracket-adhesive-enamel junction affected about 25% of patients undergoing orthodontic treatment [3]. Bacteria invading the interface between the tooth and the restorative material are the principal etiologic factor responsible for secondary caries [4–6]. For a full-coverage crown or a bridge, gingival inflammation may be irritated instead of caries [7]. Similarly, pathogenic bacteria attached on the implant surface can cause inflammation in the surrounding bone or inhibit osseointegration, which would be one of the reasons for implants failure [8].

Nowadays, the mechanisms of bacteria adhesion and colonization and biofilm formation on the biomaterials are of great interest in dental research [9]. Surface characteristics such as roughness and surface energy have been reported to be relevant to the adhesion and development of microbial biofilms [10]. On the one hand, previous studies have shown that rough surfaces tend to be favorable substrata for plaque retention by providing larger surface area and nonshedding sites, as a result facilitating bacteria colonization and biofilms maturation [11, 12]. It has been concluded that increase in surface roughness above the Ra threshold of 0.2 *μ*m facilitates biofilm formation on restorative materials. On the other hand, Mitik-Dineva et al. [13] indicated that bacteria adhesion was significantly influenced by nanometer-scale changes of surface roughness and concluded nanoscale surface roughness may be more sensitive to bacterial adhesion than was previous believed. However, Etxeberria et al. [9] claimed neither roughness or surface roughness greatly influenced bacteria attachment. Thus, a general consensus has not yet been obtained in the literature on the role of the surface roughness on bacterial attachment.

It is well known that oral biofilms are complex and dynamic microbial structures [14]. The formation of biofilm in oral cavity has four stages: transport of bacteria, initial bacterial adhesion, attachment, and biofilm maturation [12]. Previous studies have evaluated the early adhesion (30 min [15], 1 h [16, 17], 1.5 h [18], 2 h [9], 2.5 h [19], 3 h [20], 4 h [21], and 6 h [22]) on the biomaterial or focused more on the longer exposure (12 h [13], 24 h [9, 20, 23–25], 48 h [17], 2 weeks, and 3 months [26]) in microbiological environment. However, these studies mainly focused on quantification of biofilm accumulation. The process from early attachment to later formation of biofilm on zirconia regarding the impact of surface roughness is poorly elucidated. Furthermore, to our best knowledge, no study has been conducted in regard to direct adhesion forces of* Streptococcus mutans* (*S. mutans*) to zirconia with different roughness.

Atomic force microscopy (AFM) enables researchers to study the interactions between cell-cell and cell-solid surfaces [27]. It employs a cantilever that deflects in proximity of a surface to detect the adhesion forces between cantilever and solid surfaces. Biological samples, such as living cells, can also be investigated due to their ability to acquire data under liquids [28]. When the cantilever is functionalized with bacteria, AFM can be used to measure forces between bacteria and another solid surface [29].

In order to prevent the biofilm formation on the restorations, it is essential to understand the mechanisms of oral bacterial adhesion and biofilm development on the restorative biomaterials surface. The present study was to investigate the effect of zirconia surface roughness on the initial adhesion and biofilm formation of* S. mutans*, a primary pathogenic bacteria in dental caries. AFM was employed to determine the zirconia topography and the adhesion force between the bacteria and zirconia surface. In addition, the amount of bacteria colonized on the zirconia surface with diverse degree of roughness was quantified after biofilm formation. Our hypothesis is that the adhesion forces and attachment of* S. mutans* on the zirconia are dependent on the surface roughness.

## 2. Materials and Methods

### 2.1. Specimen Preparation

Zirconia round discs were purchased from Shenzhen Kangtaijian Dental Labs (Shenzhen, China), a company supplying the zirconia from Sirona Dental. The composition of zirconia samples is as follows: zirconium dioxide (ZrO_2_) and hafnium oxide (94%~95% wt), yttrium oxide (4.5%~5.5% wt), and aluminium oxide (<0.5% wt). The discs were fabricated with CEREC 3D CAD/CAM device (Sirona Dental Systems, Charlotte, NC) by cutting into cuboid blocks, followed by sintering at 1480°C for 8 h and heat preservation for 2 h before cooling. The final size of experimental zirconia blocks was strictly controlled with dimensions of 10 mm × 10 mm × 2 mm. One side of the specimen was randomly selected and polished to an initial flat surface (10 mm × 10 mm) with 180-grit silicon carbide abrasive paper (Struers, Germany) and sequentially finished with 400- (Coarse group), 600- (Medium group), or 800- (Fine group) grit silicon carbide abrasive paper under running water using Struers polishing machines at a speed of 800 rpm for 5 min for each grade. The specimens were finally ultrasonically cleaned in deionized water and then ethanol for 15 min.

### 2.2. Surface Topography Analyses

The morphological features of polished zirconia were measured by SPM-9600 AFM system (Shimadzu, Kyoto, Japan) in phase mode with a V-shaped cantilever with silicon tip of HYDRA-ALL-G-50 (a spring constant of 0.284 N/m, tip height of 4–6 *μ*m, and a tip curvature radius of <8 nm, APPNANO, USA) under ambient condition. Five randomly selected sites of each zirconia block were scanned with the scanning rate of 1.0 Hz and area of 10 *μ*m × 10 *μ*m. The average surface roughness (Ra) and 3-dimensional images were obtained and analyzed with AFM systemic software (VectorScan 3.3.1, Shimadzu).

### 2.3. Surface Wettability

The surface contact angles of zirconia blocks were determined using a contact angle goniometer (DSA30, KRÜSS, Germany) by dropping 1 *μ*L of deionized water onto each randomly selected site of polished surface. Contact angles for five chosen sites were averaged.

### 2.4. Bacterial Functionalization

The preparation of bacterial AFM tips was described as previous study [30]. Briefly,* S. mutans* UA159 was cultured in 10 mL of brain heart infusion (BHI, Oxoid, UK) broth overnight at 37°C in 70% N_2_ + 20% CO_2_ + 10% H_2_, followed by centrifugation at 1,500 ×g for 10 min and washing twice with PBS buffer. Resuspended bacteria were sonicated in ice/water bath before being immobilized on tipless triangle shaped AFM cantilevers (CSC37/Tipless, MikroMasch, USA) with length of 350 *μ*m, width of 35 *μ*m, thickness of 2.0 *μ*m, resonance frequency of 20 Hz, and force constant of 0.3 N/m. Cantilevers were first dipped in a drop of poly-L-lysine (0.1 mg/mL) (Sigma, Poole, UK) for 3 min under light microscope to create a positive charge. Subsequently, the poly-L-lysine coated cantilever was dried in air for 2 min and then immersed into bacterial suspension for 5 min to allow bacterial attachment. To remove unbound bacteria, the probes were rinsed with PBS buffer. All probes were prepared just before the experiments and used immediately.

### 2.5. Adhesion Force Measurement

Attachment force between bacteria and zirconia surface was measured with AFM under contact mode at room temperature in PBS buffer using a scan rate of 1.0 Hz and scan width of 1,500 nm. To avoid systematic errors due to local irregularities of the surface, every measurement was done on randomly chosen 5 spots on the zirconia surfaces. Ten force-distance curves were obtained for each single spot. SEM was regularly proceeded to confirm the integrity of the bacteria probe on the functionalized cantilever after measurement. The force curves would be disposed once the bacteria layer was damaged.

### 2.6. Biofilm Formation Assay

An initial suspension of* S. mutans* with OD600 of 0.5 was prepared by diluted overnight cultured* S. mutans* with BHI. Two milliliters of the 250-fold dilution of initial bacteria suspension with 1% sucrose was added into each well of 24-well polystyrene plates. One sterilized zirconia block with polished surface upward was placed in each well and incubated at 37°C in 70% N_2_ + 20% CO_2_ + 10% H_2_ for 2 h, 4 h, 6 h, 8 h, 12 h, or 24 h.

### 2.7. Acridine Orange Stain

To test the early attached bacteria on zirconia surface, the specimens with incubation in bacteria suspension for 2 h were removed and gently rinsed with PBS twice and then left to dry in laminar flow cabinet. Cells retained on the surface were stained with acridine orange (1% in distilled water). The samples were rinsed and dried before examination with epifluorescence microscopy at 400x magnification (OLYMPUS BX3, Japan). Five fields of each surface were examined. The pictures were taken by software Andor and added color by software Cell Sens Dimension. The number of cells in each filed was counted using Image Pro Plus.

### 2.8. MTT Assay

To quantify adherent biomass of* S. mutans* biofilm, the MTT assay was performed. After incubation for 4 h, 8 h, 12 h, or 24 h, the zirconia blocks with* S. mutans* biofilm were rinsed by immersion in PBS twice to remove loosely bound bacteria and transferred to a new 24-well plate, followed by incubating with 1 mL of 0.5 mg/mL of MTT solution (Sigma-Aldrich, USA) in PBS at 37°C for 30 min in the dark. The MTT was discarded before incubating with 1 mL of DMSO for 10 min at room temperature at 100 rpm in the dark. After pipette mixing, 200 *μ*L of mixture was transferred to a 96-well plate in triplicate. The absorbance of 570 was measured immediately with Microplate spectrophotometer (Multiskan, Thermo Fisher).

### 2.9. Statistical Analyses

The mean and standard deviation of surface roughness (*n* = 6), contact angles and surface energy (*n* = 7), adhesion strength (60 curves), acridine orange stain (*n* = 4), and biofilm attached (*n* = 6) were analyzed using the one-way analysis of variance (One-way ANOVA) and Tukey's multiple comparison test with SPSS 16.0 statistical software (SPSS Inc., USA). The level of statistical significance was set at *P* < 0.05.

## 3. Results

### 3.1. Surface Topography

**Figure 1:**
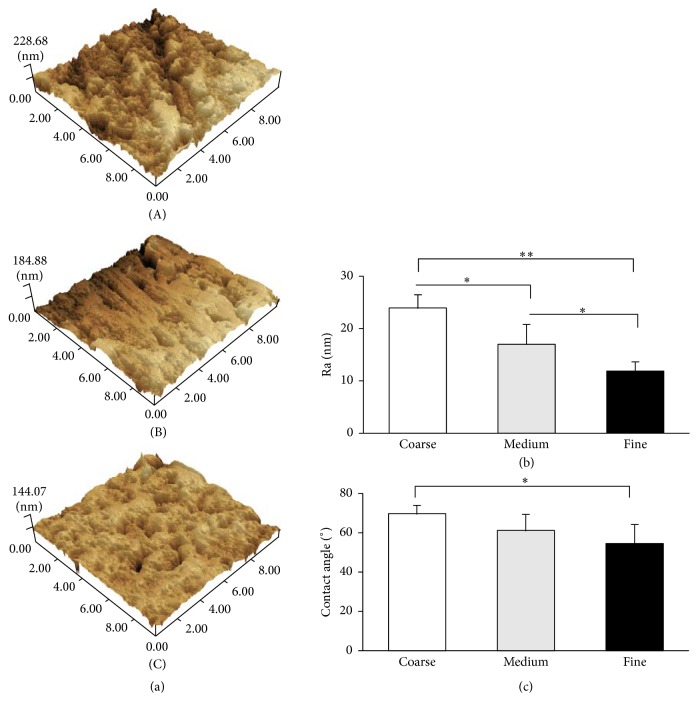
Surface properties. (a) Representative 3D images of zirconia surface morphology in Coarse (A), Medium (B), and Fine (C) groups. (b) Mean values (nm, ±SD) of the surface roughness (Ra) of three groups. (c) Mean contact angles. ^*∗*^ *P* < 0.05 and ^*∗∗*^ *P* < 0.001.

The surface morphology and roughness were characterized by AFM (Figure 1(a)). The scratches in Coarse and Medium groups were obvious to visualize by AFM, but the scratched area of the Medium group was less than the Coarse group. The surface of Fine specimen was smoother compared to Coarse and Medium. One-way ANOVA showed significant differences on Ra values among tested groups (*P* < 0.05). Tukey's multiple comparison test revealed that the surface roughness of the Coarse group (23.94 ± 2.52 nm) was significantly higher than the Medium (17.00 ± 3.81 nm) and the Fine groups (11.89 ± 1.68 nm), while the Medium group had rougher surface compared to the Fine group (*P* < 0.05) (Figure 1(b)).

### 3.2. Contact Angles

The mean values of water contact angles were reported in Figure 1(c). The Coarse zirconia was the most hydrophobic, with a contact angle of 69.05 ± 4.00, followed by the Medium surface (60.54 ± 7.46) and Fine surface (56.38 ± 10.13). There was significant difference between Coarse and Fine groups (*P* < 0.05); however, the Medium group (60.54 ± 7.46) had statistical difference neither with Coarse group nor with Fine group.

### 3.3. Bacterial AFM Tip

**Figure 2:**
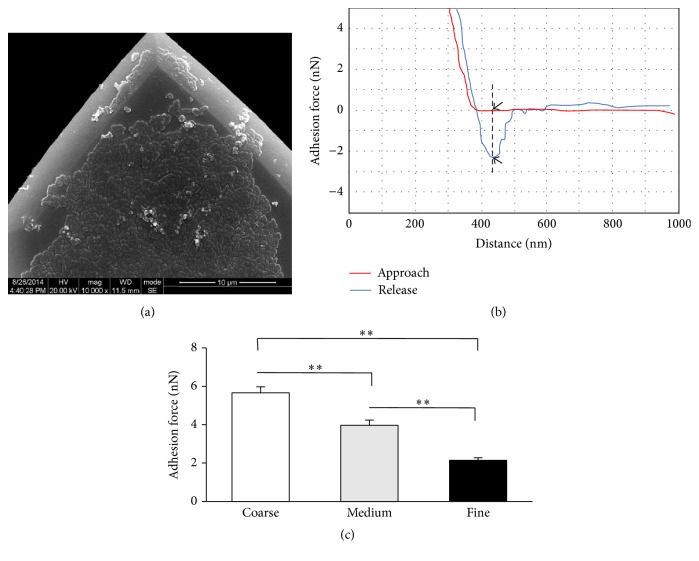
Adhesion force. (a) SEM for bacteria functionalization (×10000). (b) Typical force-distance curve between* S. mutans* and zirconia surface, the value of adhesion force was the vertical difference between two arrows. (c) The mean values of adhesion force from three groups. ^*∗∗*^ *P* < 0.001.

In order to make sure the AFM tips were functionalized with bacteria, SEM was employed (Figure 2(a)), demonstrating bacteria cells were successfully attached on the AFM tip.

### 3.4. Adhesion Force

A typical force curve is shown in Figure 2(b). The maximum adhesion force was recorded as the difference between the adhesion peak and the baseline and the vertical distance between two arrows was marked in the graph. The mean values of adhesion strength between the bacteria and zirconia surface are illustrated in Figure 2(c), indicating significant difference in the adhesion force among three groups (*P* < 0.001). The Coarse group had the largest adhesion force, whereas the Fine group had the lowest. Positive correlation between surface roughness and initial adhesion force was observed (Figure 5(a), *Y* = 0.2892*X* − 1.165, *R* ^2^ = 0.9880).

### 3.5. Acridine Orange Stain

**Figure 3:**
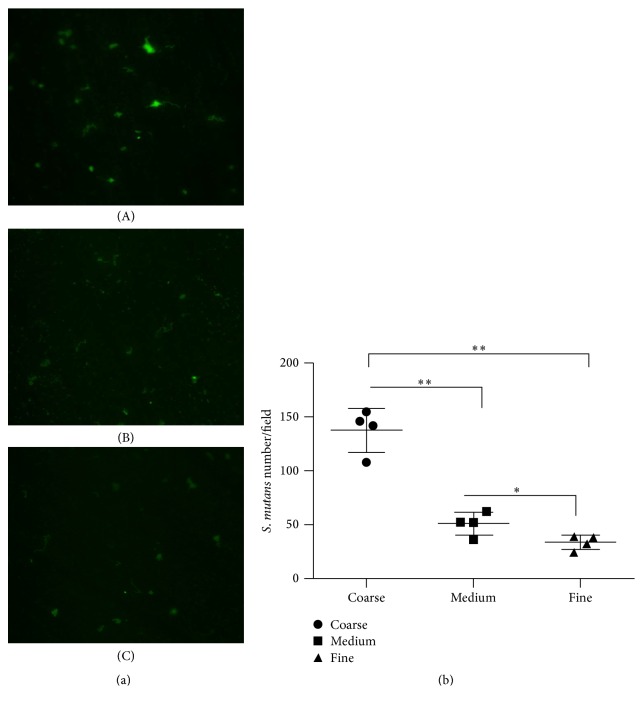
The acridine orange stain. (a) The acridine orange stain of* S. mutans* on zirconia surface in Coarse (A), Medium (B), and Fine (C) groups after incubation for 2 h. (b) The number of attached* S. mutans* on the zirconia surfaces (mean ± SD). ^*∗*^ *P* < 0.05 and ^*∗∗*^ *P* < 0.001.

The images of acridine orange staining for early attached bacteria were displayed in Figure 3(a). In accordance with the result of adhesion force, the number of attached bacteria in each field in Coarse group was the largest compared to other groups, while the Fine group had the lowest number of early attached bacteria. Likewise, positive correlation was determined between surface roughness and the number of early attached bacteria (Figure 5(b), *Y* = 8.898*X* − 82.8, *R* ^2^ = 0.9228).

### 3.6. MTT Assay

**Figure 4:**
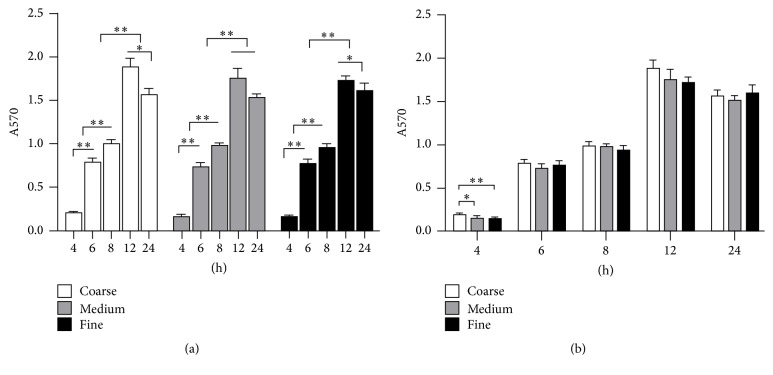
Bacterial accumulation on the zirconia surface after incubation for 4 h, 6 h, 8 h, 12 h, and 24 h. (a) Variation at different time points in respective groups. (b) Comparison among Coarse, Medium, and Fine groups at indicated time points. ^*∗*^ *P* < 0.05 and ^*∗∗*^ *P* < 0.001.

**Figure 5:**
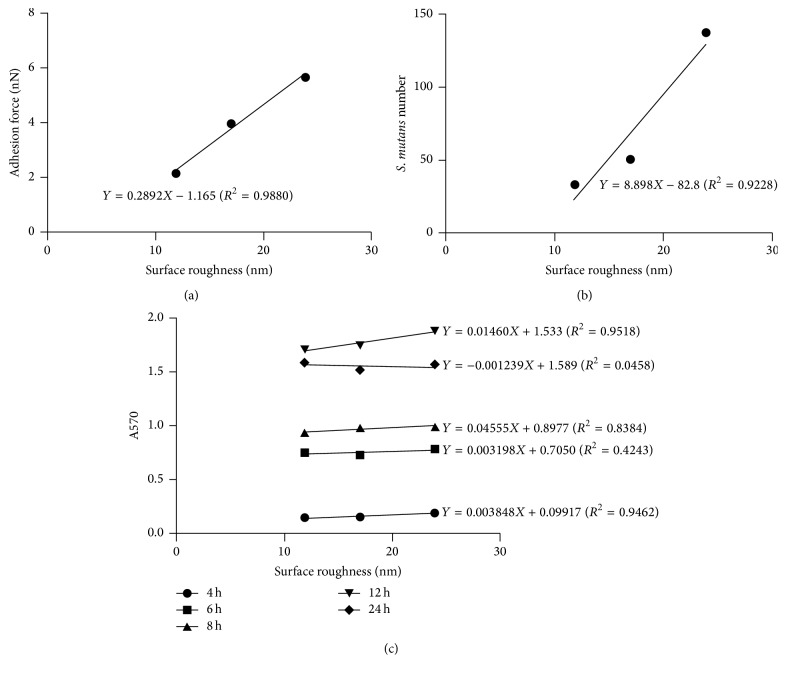
Correlation between the surface roughness and the* S. mutans* attachment. (a) The correlation between the surface roughness and the initial adhesion force. (b) The correlation between the surface roughness and the number of attached* S. mutans* after incubation for 2 h. (c) The correlation between the surface roughness and A570 after incubation for 4 h, 8 h, 12 h, and 24 h.

The amount of colonized* S. mutans* increased with incubation time increasing from 4 h to 12 h (*P* < 0.001) and then decreased at 24 h (Figure 4). Similarly to 2 h, the results of* S. mutans* colonization among three tested groups at 4 h had significant difference. However, thus difference could not be observed in the following time points. The correlation between the surface roughness and the amount of colonized bacteria was positive from 4 h to 12 h, whereas weak negative correlation was observed at 24 h (Figure 5(c)).

## 4. Discussion

ZrO_2_ is becoming a favorable material in restorative dentistry. In clinic, chair-side modifications and adjustments for the zirconia restorations are sometimes inevitable in order to achieve an optimal interproximal contact condition and occlusal relationship [31]. These procedures are usually performed by grinding the ceramic surface which may cause loss of the glazed layer and result in roughened surfaces despite following sequential polish [32]. A number of investigations presented the notion that the roughness of surface has a major impact on the initial adhesion and accumulation of bacteria, contributing to some oral diseases, such as caries and periodontal inflammation. However, contradictory effects of surface roughness on the bacteria adhesion and/or biofilm formation have been reported. Therefore, the current study evaluated the relation between* S. mutans* colonization and the zirconia surface properties.

In this study, in attempt to produce surfaces with various morphologies, the specimens were polished in standardized manner. Although 800-, 600-, 400-, or 180-grit silicon carbide abrasive papers are commonly not used to polish zirconia restorations in clinic, adopting those abrasive papers in this study was to obtain the flat, uniform surfaces without undulations. Our results of AFM confirmed there were statistically significant differences in Ra values of the Coarse, Medium, and Fine groups, and the Ra values of all the groups were nanoscale (under 30 nm).

As we all know, the formation of biofilm is a complicated multistep process initiated by reversible attachment of bacteria to surfaces [33]. In present study, we tested the adhesion force between the bacteria and zirconia surface with different rough condition using AFM, which was considered more related to initial adhesion for the bacteria since the approaching process of AFM tips with bacteria to contact with zirconia surface was similar to the reversible adhesion phase. Both processes have quite a short period of time and an unstable interaction between bacteria and surface. According to the Derjaguin–Landau–Verwey–Overbeek (DLVO) theory [34], initial adhesion involves a number of unspecific interactions, namely, van der Waals attractive forces (vdW) and electrostatic repulsive forces. The vdW are the predominant forces while the distances between microorganism and the surface were greater than 50 nm, whereas the combination of vdW and electrostatic interactions governed the bacteria adhesion when at closer distance [35]. Our results showed that nanoscale roughness has positive effect on the* S. mutans* adhesion forces. The strongest bacterial adhesion forces were detected in the Coarse group, while adhesion to the finer surfaces was weaker. This probably could be explained by the notion that the contacting area of rougher surface is larger than flat surface since the bacteria cells could be pressed into the grooves during approaching process of cantilever due to the elastic deformability of the cells [30].

However, the adhesion force can only represent the initial stage of biofilm formation, not including the next coming processes. In fact, the initial bacteria adhesion was preceded by the early attachment stage and later maturation stage. Therefore, we determined the subsequent biofilm formation by quantifying the amount of accumulated bacteria on the zirconia surface. Several investigations have suggested that the initial adherence occurs at defects on the surfaces such as grooves; then the accumulation of bacteria spreads out from the irregularities to other areas; they concluded a threshold surface roughness of Ra = 0.2 *μ*m for bacterial retention [11]. Based on this point of view, Ra ≤ 0.2 *μ*m had a negligible influence on bacterial adhesion while, over this value, bacteria accumulation increased with increasing roughness. However, our findings suggested that even nanoscale roughness positively affects the bacteria colonization. Similarly, the minimum level of surface roughness has been questioned and the impact of nanometer-scale roughness on bacterial attachment has been investigated recently [13, 17, 18, 36–38]. Mitik-Dineva et al. [13] suggested that bacteria may be more sensitive to nanoscale surface roughness than was previously believed. Yoda et al. [17] indicated that the amount of bacteria that adhered to five kinds of biomaterials (oxidized zirconium-niobium alloy, cobalt-chromium-molybdenum alloy, titanium alloy, commercially pure titanium, and stainless steel) with rougher surfaces (7.2 nm ≤ Ra ≤ 30 nm) was greater than those with smoother surfaces (1.8 nm ≤ Ra ≤ 8.5 nm). Lee et al. [18] demonstrated that there is no significant difference in bacterial adherence capability between titanium (Ra = 0.059 *μ*m) and zirconia (Ra = 0.064 *μ*m), but significantly high amounts of bacteria adhered to resin (Ra = 0.179 *μ*m). Xing et al. [36] evaluated oral biofilm formation with nanoscales surfaces (29 nm~214 nm) using a splint model* in vivo* and found that nanoroughness positively correlated with bacteria adhesion.

Apart from providing extended surface for initial adhesion, a rough surface also increases the surface area available for bacteria accumulation compared to a smooth surface. It was found both* in vivo* and* in vitro* that bacteria accumulated to a greater degree on rough surfaces than on a highly polished surface [11, 12, 20]. In current study, the rougher surface tended to have higher attraction to bacteria than smoother surface but only in the early stages (2 h and 4 h) of attachment. Bacteria prefer to anchor on rough surfaces since they are better sheltered against displacing shear forces, thus having enough time to transit from reversible to irreversible adhesion and as a result biofilms are able to mature [12]. However, after 6 h of incubation, surface roughness appeared to have no influence on the amount of adherent bacteria. This phenomenon corresponds with some former studies [25, 39, 40]. Morgan and Wilson [39] indicated that the roughness of acrylic can only affect the early stages of biofilm formation by* S. oralis in vitro*. do Nascimento et al. [25] found that there was no significant difference in the total area of biofilm formed on the intraoral splint for 24 h* in vivo*. A possible reason is that the influence of roughness and material on biofilm formation was compensated by the proceeding maturation of the oral biofilm [40]. These results suggested that bacterial adhesion forces and early proliferation positively correlated with surface roughness.

In current study, not only had the surface roughness been considered, but also the hydrophobicity (contact angles) of the zirconia specimen was investigated. Hydrophobicity also plays a key role in bacterial adhesion and biofilm formation efficiency. Etxeberria et al. [9] studied the attachment of* E. coli* and* S. aureus* on various dental implant materials and found marked difference of* E. coli* attachment depending on the materials after 2 h whereas similar differences were not observed for* S. aureus*. After 24 h, both species no longer had significant difference. They considered that neither roughness nor nanoroughness affected bacteria attachment; instead wettability strongly correlated with adhesion, suggesting that the bacterial adhesion could not count on the roughness alone. Our results have shown that surface roughness positively influenced hydrophobicity and the more hydrophobic surface (Coarse group) exhibited greater colonized bacteria. This might be explained by the fact that hydrophobic surfaces tend to attract the accumulation of proteins which provide specific binding sites for bacteria, accelerating and facilitating bacterial adhesion [41]. However, alteration in surface roughness will in most cases also alter the surface energy. It is therefore difficult to distinguish between the two factors.

There are some studies which suggested that the correlation between surface properties and bacteria adhesion was poor. Zhao et al. [42] concluded that neither roughness nor hydrophobicity decisively affected biofilm formation. Hahnel et al. [19] conducted research on the surface characterization of dental ceramic and the correlation of initial adherence of three oral streptococcal strains, but no correlation has been observed. Discordance may be derived from a number of different factors relating to both bacteria and substratum. Pita et al. studied the behavior to form biofilm of five oral streptococci species on various dental implant surface topographies. Their data showed that* S. cricetus*,* S. mutans*, and* S. sobrinus* exhibited higher biofilm formation compared to* S. salivarius* and* S. sanguinis*, suggesting that biofilm formation depends on not only the surface topography but also the bacteria species involved [43]. Similarly, Mei et al. [44] reported that although both* S. sanguinis* and* S. mutans* were sensitive to changes in surface roughness, the initial adhesion forces of the former, initial colonizer, were more affected. Cell shape and size, surface characteristics such as wettability, extracellular substances such as flagella and fimbriae, and production of proteins are believed to have a significant influence on the process of bacteria adhesion [45]. Furthermore, surface roughness is scale-dependent and can be measured in many approaches; therefore, the values of surface roughness parameters may be different at the macroscale compared to the microscale and even at the nanoscale [46]. According to Preedy et al., the correlation between surface roughness and adhesion forces varies as the size of the area is scanned to calculate roughness value [35].

As expected, the amount of bacteria adhered on the zirconia surface increased with incubation time. Interestingly, the amount of colonized* S. mutans* kept increasing by 12 h but decreased at 24 h. This might suggest that the detachment of bacteria from the biofilm and dispersal occurred in the final period.

The data of present study support the hypothesis that the adhesion forces and attachment of* S. mutans* on the zirconia are dependent on the surface roughness. Due to chipping of the veneering layer, the most common reported clinical complication of ceramic restoration, full-contour zirconia (FZ) restorations were suggested to be produced as an alternative to solve this problem [32, 47]. The FZ restorations are glazed in most cases; however, unavoidable adjustments may expose the zirconia to the oral environment where more than 600 types of different bacteria dwell. Thus, several pathogenic processes may occur around the restorations. This emphasizes the importance of polishing and finishing of zirconia restorations after adjustment in clinic.

## 5. Conclusions

In conclusion, we compared the adherence capability of* S. mutans* to zirconia surfaces with three different levels of roughness in nanoscale and determined the attachment activities of* S. mutans* from very early adhesion to further biofilm formation. Within the limitations of this research, we concluded that the surface nanoroughness and hydrophobicity of zirconia had influence on not only the adhesion forces but also the early attachment of* S. mutans*. The current study deepened our understanding on the mechanism of bacterial adhesion and biofilm formation on the dental restorative materials. More researches* in vitro* and* in vivo* are needed with respect to other oral bacteria and factors that may affect the results.
